# Factors associated with HIV testing among youth aged 15–24 years in Myanmar: evidence from the 2015–16 demographic and health survey

**DOI:** 10.3389/frph.2026.1804929

**Published:** 2026-05-04

**Authors:** Soe Sandi Tint, Myo Zin Oo

**Affiliations:** 1 Global Health and Chronic Conditions Research Center, Chiang Mai University, Chiang Mai, Thailand; 2 Department of Family Medicine, Faculty of Medicine, Chiang Mai University, Chiang Mai, Thailand; 3 Research Institute for Health Sciences, Chiang Mai University, Chiang Mai, Thailand

**Keywords:** demographic and health survey, HIV prevention, HIV testing, Myanmar, sexual and reproductive health, youth

## Abstract

**Introduction:**

HIV testing is central to early diagnosis and HIV prevention, yet uptake among youth remains suboptimal in many low- and middle-income countries. Our study examined HIV testing uptake and its associated factors among youth aged 15–24 years in Myanmar.

**Methods:**

We conducted a cross-sectional analysis of the 2015–16 Myanmar Demographic and Health Survey (MDHS), restricting the sample to youth aged 15–24 years (*N* = 5,185). Survey-weighted descriptive statistics and bivariable and multivariable logistic regression analyses were used to examine factors associated with HIV testing, with results reported as adjusted odds ratios (AORs) and 95% confidence intervals (CIs). Statistical significance was defined as *p* < 0.05, with analyses accounting for the complex sampling design.

**Results:**

Overall, 10.9% of youth reported ever having been tested for HIV. In multivariable analyses, youth aged 20–24 years had higher odds of HIV testing than those aged 15–19 years (AOR = 2.57; 95% CI: 1.94, 3.39; *p* < 0.001), and males had higher odds than females (AOR = 1.66; 95% CI: 1.27, 2.18; *p* < 0.001). Youth who had ever had sexual intercourse were substantially more likely to have been tested (AOR = 4.59; 95% CI: 3.54, 5.96; *p* < 0.001).

**Conclusion:**

HIV testing uptake among youth in Myanmar remains low, with testing concentrated among older and sexually experienced youth. Strengthening youth-friendly HIV testing strategies that proactively reach adolescents and socioeconomically disadvantaged youth is critical to improving early diagnosis and advancing HIV prevention and youth sexual and reproductive health in Myanmar.

## 1 Introduction

Human immunodeficiency virus (HIV) represents a substantial public health burden worldwide and continues to disproportionately affect adolescents and young people (1, 2). In 2019, an estimated 38 million people were living with HIV (PLHIV) worldwide, yet approximately 19% were unaware of their HIV status (3), highlighting persistent gaps in HIV testing coverage. Early HIV diagnosis through testing plays a central role in HIV prevention and treatment by enabling early initiation of antiretroviral therapy (ART), reducing onward transmission, and improving long-term health outcomes (4, 5). From a sexual and reproductive health perspective, HIV testing provides an important pathway for adolescents and young adults to access prevention and care services. Among adolescents and young adults, social and behavioral barriers contribute to delayed or limited uptake of HIV testing (6). However, despite expanded testing services and global progress in HIV control, evidence indicates that HIV testing among young people remains insufficient in many low- and middle-income countries (7, 8).

Myanmar is among the countries with a high HIV burden, with the epidemic concentrated in key populations such as people who inject drugs, female sex workers, and men who have sex with men (MSM) (9, 10). Between 2000 and 2019, annual new HIV infections in Myanmar declined substantially, reflecting progress in HIV prevention and treatment efforts (3). In 2017, an estimated 224,000 people were living with HIV, with a national prevalence of approximately 0.57% (9). By 2022, this number was estimated to have increased to around 280,000 PLHIV, while Myanmar had achieved high levels of antiretroviral therapy coverage and viral suppression, with 94% of those receiving treatment achieving viral suppression (10). These epidemiological patterns indicate that, despite progress in treatment and reductions in new infections, HIV remains an important public health challenge in Myanmar.

Youth aged 15–24 years constitute a key population in the HIV response, as this stage of life is marked by increased vulnerability linked to rapid social and behavioral change. Shifts in educational status, work participation, and sexual experience during this period may shape both vulnerability to HIV and patterns of health-care use. Previous research from diverse settings suggests that HIV testing among young people is shaped by a combination of sociodemographic factors, sexual experience, HIV-related knowledge, and exposure to health information through mass media (11). Understanding how these factors interact to influence HIV testing uptake among youth is essential for informing targeted and equitable testing strategies in settings with generalized and concentrated HIV epidemics.

Despite the importance of HIV testing for early diagnosis and prevention, nationally representative and youth-focused analytical evidence on factors associated with HIV testing uptake in Myanmar remains limited. Previous DHS-based analyses in Myanmar and Southeast Asia have primarily focused on women of reproductive age and cross-country comparisons (12, 13), with limited attention to adolescents and young people as a distinct population and to youth-specific sexual behavioral factors. To address this gap, we examined HIV testing uptake among youth aged 15–24 years in Myanmar and identified factors associated with HIV testing.

## 2 Methods

### 2.1 Study design, data source, and participants

Our study employed a cross-sectional analytical design using secondary data from the Myanmar Demographic and Health Survey (MDHS) 2015–16 (14), the most up-to-date nationally representative household survey conducted across Myanmar.

The MDHS 2015–16 was designed to provide reliable population-level estimates of key demographic and health indicators at the national, state/region, and urban-rural levels. The original MDHS survey (14) targeted women and men aged 15–49 years who were either usual residents of selected households or visitors who had stayed in the household the night before the survey. For the present analysis, we restricted the study population to youth aged 15–24 years, consistent with international definitions of adolescents and young people. All eligible respondents within this age range who had available information on HIV testing status and relevant explanatory variables were included. No additional exclusion criteria were applied.

### 2.2 Sample size and sampling

The MDHS 2015–16 (14) applied the standard DHS two-stage stratified cluster sampling design to produce representative estimates for Myanmar overall, urban and rural areas, each of the 14 states/regions, and Nay Pyi Taw Union Territory. The sampling frame was based on the 2014 Myanmar Population and Housing Census cartographic frame and included 76,990 primary sampling units (PSUs). In the first stage, 442 clusters were selected across 30 strata (urban and rural within each state/region), including 123 urban and 319 rural clusters. In the second stage, 30 households per cluster were selected using systematic sampling, yielding 13,260 households (3,690 urban; 9,570 rural). Household lists were updated prior to selection, and no household replacement was permitted. All eligible women aged 15–49 years in selected households were interviewed, while the men's survey was conducted in half of selected households. As a result, the sample includes a lower proportion of males; however, sampling weights were applied to ensure nationally representative estimates. In the achieved sample, 12,500 households were successfully interviewed (response rate 97.8%), with 12,885 women (95.8% of eligible women) and 4,737 men (90.8% of eligible men) completing interviews.

**Figure 1:**
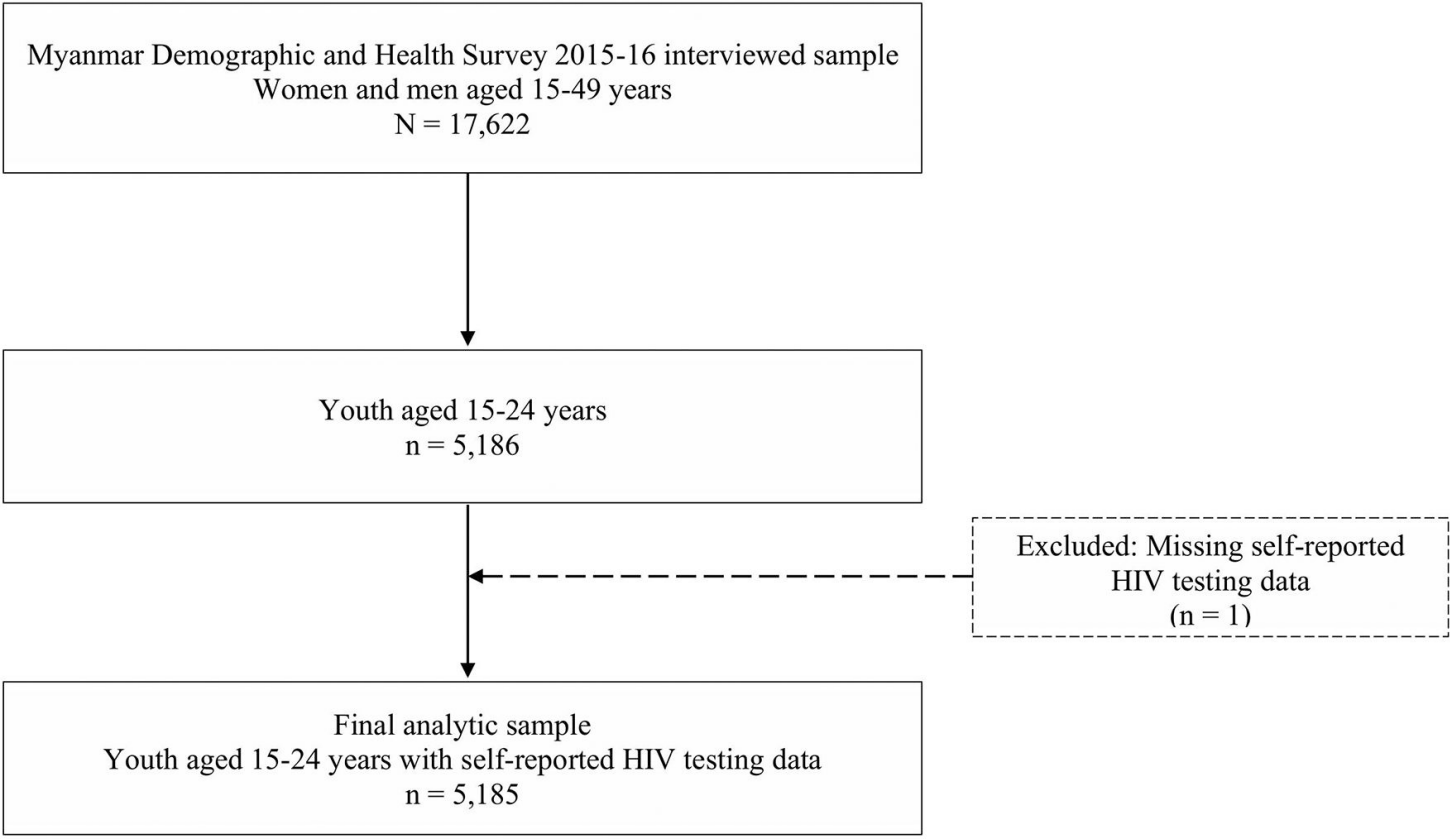
Study flow diagram among 17,622 women and men aged 15–49 years interviewed in the 2015–16 Myanmar Demographic and Health Survey, 5,186 were youth aged 15–24 years. Of these, one respondent was excluded due to missing self-reported HIV testing information. The final analytic sample therefore comprised 5,185 youth aged 15–24 years with complete HIV testing data.

For our present analysis, the study population was first restricted to youth aged 15–24 years (*n* = 5,186). One respondent was excluded due to missing self-reported HIV testing information, yielding a final analytic sample of 5,185 youth (Figure 1). All analyses were conducted using appropriate sampling weights to account for the complex survey design.

### 2.3 Training and data collection process

The MDHS 2015–16 (14) was implemented by the Ministry of Health and Sports (MoHS) with technical support from ICF through The DHS Program. Survey staff received standardized training in questionnaire administration, interviewing techniques, and field procedures, including supervised field practice. Data were collected through face-to-face interviews by trained field teams under ongoing supervision and quality control. Fieldwork was conducted nationwide from December 7, 2015, to July 7, 2016.

### 2.4 Measurements

The MDHS 2015–16 (14) employed three standardized survey instruments: the *Household Questionnaire*, the *Woman's Questionnaire*, and the *Man's Questionnaire*, developed under the global DHS program and adapted to the Myanmar context. Our present analysis used data from the women's and men's questionnaires.

### 2.5 Outcome variable

The outcome variable was HIV testing uptake, defined as self-reported ever having been tested for HIV.

### 2.6 Predictor variables

#### 2.6.1 Sociodemographic characteristics

Sociodemographic variables included age, sex, educational attainment, marital status, place of residence, occupation, household wealth quintile, and exposure to mass media.

#### 2.6.2 Sexual behavior indicators

Sexual behavior was assessed using three MDHS-derived indicators (14): ever had sexual intercourse (never vs. ever), recent sexual activity (sexually active in the four weeks preceding the survey vs. not), and age at first sexual intercourse. Age at first sex was categorized as never had sex, <18 years, or ≥18 years, allowing inclusion of all respondents and differentiation between early and later sexual debut.

#### 2.6.3 Comprehensive knowledge of HIV

Comprehensive knowledge of HIV was measured using five standard MDHS items (14) covering condom use, having one faithful uninfected partner, recognition that a healthy-looking person can have HIV, and rejection of the misconceptions that HIV can be transmitted through mosquito bites and by sharing food. Responses were coded as correct or incorrect, with “don’t know” treated as incorrect. Respondents answering all five items correctly were classified as having composite comprehensive knowledge of HIV (“Yes”); all others were classified as “No”, consistent with the standard DHS definition to ensure comparability across studies.

#### 2.6.4 HIV-related attitudes toward people living with HIV (PLHIV)

Attitudes toward PLHIV were measured using four standard DHS items (14) assessing willingness to care for a family member with HIV, buy food from a vendor living with HIV, allow an HIV-positive teacher to continue teaching, and accept disclosure of HIV status within the family. Affirmative responses indicating acceptance were coded as non-discriminatory, while negative responses were coded as discriminatory; uncertain responses were treated as missing. Respondents expressing non-discriminatory attitudes across all four items were classified as having a composite non-discriminatory attitude toward PLHIV.

### 2.7 Statistical analysis

Data analysis was conducted using STATA version 15. Descriptive statistics, including frequencies and weighted percentages, were used to summarize explanatory variables and HIV testing uptake. The outcome variable had no missing data; however, some independent variables had missing values, and analyses were conducted using complete cases for each model. Bivariate associations between explanatory variables and HIV testing were assessed using chi-squared tests, with detailed results presented in the Supplementary Table S1. Variables associated with HIV testing in bivariate analyses (*p* < 0.25) were considered for inclusion in multivariable models. Bivariable (unadjusted) and multivariable (adjusted) logistic regression analyses were performed to examine factors associated with HIV testing uptake (ever tested vs. never tested). Results are reported as odds ratios (ORs) and adjusted odds ratios (AORs) with 95% confidence intervals (CIs). Final model specification was guided by theoretical relevance, prior evidence, and assessment of multicollinearity. Given the strong conceptual overlap among sexual behavior and related variables (e.g., marital status, recent sexual activity, and age at first sexual intercourse) among youth, only one representative sexual behavior variable (ever had sexual intercourse) was retained in the final multivariable model to minimize redundancy and potential overadjustment.

Multicollinearity was assessed using variance inflation factors (VIFs) derived from an equivalent linear regression model. The mean VIF was 2.42, and no evidence of problematic multicollinearity was observed. Overall model significance was assessed using a survey-adjusted Wald *F* test, which indicated that the multivariable model fit the data significantly better than the null model (F = 18.09, *p* < 0.001). Statistical significance was defined as *p* < 0.05.

All analyses accounted for the complex two-stage stratified cluster sampling design of the MDHS by applying sampling weights, clustering, and stratification using the “svyset” command in STATA. This approach accounts for unequal selection probabilities and clustering, ensuring nationally representative and unbiased estimates in both descriptive and regression analyses (15). Unweighted cell counts were examined, and some variable subgroups contained fewer than 25 observations; these estimates were retained but interpreted with caution due to potential instability.

### 2.8 Ethical considerations

The MDHS 2015–16 (14) was conducted in accordance with established ethical standards. The survey protocol was reviewed and approved by the Ethics Review Committee on Medical Research including Human Subjects, Department of Medical Research, Ministry of Health and Sports, Myanmar, and by the ICF Institutional Review Board. All participants provided informed consent, and respondent confidentiality was strictly maintained.

Our present study involved a secondary analysis of de-identified MDHS data. Permission to access and use the dataset was obtained from The DHS Program (https://dhsprogram.com/), with initial approval granted on October 9, 2023, and updated approval provided on January 13, 2026 following a revision of the project title in accordance with DHS data-use requirements. As the analysis used publicly available, anonymized data, no additional ethical approval was required.

## 3 Results

Table 1 presents the sociodemographic characteristics of the respondents. The analytic sample comprised 5,185 youth aged 15–24 years, with an approximately equal distribution between those aged 15–19 years (49.8%) and 20–24 years (50.2%). Females accounted for most respondents (72.1%). Most participants had completed secondary education (58.4%), resided in rural areas (69.3%), and were never married (72.1%). Nearly one-third of respondents were not working (29.2%), household wealth was relatively evenly distributed across quintiles, and a large proportion of youth reported high exposure to mass media (73.9%).

**Table 1:** Sociodemographic characteristics of the respondents (analytic sample, *N* = 5,185).

Variables	Unweighted Number(*N*)	Weighted Percentage (%)	Weighted 95% CI
Age			
15–19	2,602	49.8	48.2, 51.4
20–24	2,583	50.2	48.6, 51.8
Gender			
Female	3,727	72.1	70.6, 73.6
Male	1,458	27.9	26.4, 29.4
Education (*n* = 5,184)			
No education	338	7.3	5.7, 9.3
Primary	1,308	26.5	24.4, 28.7
Secondary	3,118	58.4	55.9, 60.9
Higher	420	7.8	6.7, 9.1
Residence			
Urban	1,538	30.7	28.4, 33.0
Rural	3,647	69.3	67.0, 71.6
Marital status			
Never married	3,744	72.1	70.3, 73.9
Married	1,339	25.9	24.3, 27.8
Not currently married or cohabiting^a^	102	2.0	1.6, 2.4
Occupation (*n* = 5,172)			
Not working	1,569	29.2	26.9, 31.4
Agriculture/ Self-employed	782	15.4	12.9, 18.4
Clerical/ sales/ services	706	13.8	12.3, 15.5
Professional/ technical/managerial	299	4.9	3.9, 6.1
Skilled manual	607	12.7	10.9, 14.7
Unskilled manual	1,209	24.0	21.5, 26.7
Wealth quintile			
Lowest	928	17.8	15.7, 20.1
Second	961	18.0	16.2, 19.8
Middle	1,158	21.5	19.5, 23.7
Fourth	1,126	21.6	19.5, 23.8
Highest	1,012	21.1	18.6, 23.9
Exposure to mass media			
No exposure	523	10.2	8.4, 12.3
Low exposure	915	15.9	14.6, 17.4
High exposure	3,747	73.9	71.6, 76.1

a Includes widowed, divorced, and separated/no longer living together.

Numbers (*N*) are unweighted counts, and percentages (%) are weighted to represent the national population with 95% confidence intervals (CI). Denominators may vary slightly due to item nonresponse.

Table 2 summarizes sexual behavior, comprehensive HIV knowledge, and HIV-related attitudes toward people living with HIV among respondents. Overall, 29.2% of youth reported ever having had sexual intercourse, and 22.1% were sexually active in the four weeks preceding the survey. Early sexual debut was relatively uncommon, with 9.6% reporting first sexual intercourse before age 18, while 18.3% initiated sexual activity at age 18 or older. Comprehensive HIV knowledge remained limited, with only 18.5% of respondents demonstrating correct knowledge across all five items. Moreover, most of the youth (83.4%) expressed discriminatory attitudes toward PLHIV.

**Table 2:** Sexual behavior, comprehensive HIV knowledge, and HIV-related attitudes toward PLHIV among respondents (analytic sample, *N* = 5,185).

Variables	Unweighted Number (*N*)	Weighted Percentage (%)	Weighted 95% CI
Ever had sexual intercourse (*n* = 5,143)			
Never	3,640	70.8	68.9, 72.5
Ever	1,503	29.2	27.5, 31.1
Recent sexual activity (last 4 weeks) (*n* = 5,143)			
Not currently sexually active	4,074	77.9	76.3, 79.5
Currently sexually active	1,069	22.1	20.5, 23.7
Age at first sexual intercourse (*n* = 5,087)			
Never had sex	3,640	72.1	70.3, 73.8
<18 years	507	9.6	8.5, 10.8
≥18 years	940	18.3	17.0, 19.7
Comprehensive knowledge of HIV (*n* = 4,657)			
No	3,800	81.5	79.9, 82.9
Yes	857	18.5	17.1, 20.1
HIV-related attitudes toward PLHIV (*n* = 4,657)			
No discriminatory attitude	771	16.6	15.1, 18.3
Discriminatory attitude	3,886	83.4	81.7, 84.9

Numbers (*N*) are unweighted counts, and percentages (%) are weighted to represent the national population with 95% confidence intervals (CI). Denominators may vary slightly due to item nonresponse.

Table 3 presents HIV testing uptake among youth aged 15–24 years. Overall, only 10.9% of respondents reported ever having been tested for HIV, while the vast majority (89.1%) had never undergone HIV testing.

**Table 3:** HIV testing uptake of the respondents (analytic sample, *N* = 5,185).

HIV testing uptake	Unweighted Number(*N*)	Weighted Percentage (%)	Weighted 95% CI
No	4,546	89.1	88.0, 90.1
Yes	639	10.9	9.9, 12.0

Numbers (n) are unweighted counts, and percentages (%) are weighted to represent the national population with 95% confidence intervals (CI).

Table 4 presents factors associated with HIV testing uptake among youth aged 15–24 years. In the multivariable analysis, youth aged 20–24 years had significantly higher odds of HIV testing compared with those aged 15–19 years (AOR = 2.57; 95% CI: 1.94, 3.39, *p* < 0.001), and male youth were more likely to have been tested than females (AOR = 1.66; 95% CI: 1.27, 2.18, *p* < 0.001). Youth who reported ever having had sexual intercourse showed markedly higher odds of HIV testing (AOR = 4.59; 95% CI: 3.54, 5.96, *p* < 0.001). Youth in the middle, fourth, and highest wealth quintiles had higher odds of HIV testing than those in the lowest quintile (*p* = 0.046, 0.004, and 0.003, respectively). In contrast, residence, higher education, comprehensive HIV knowledge, and HIV-related attitudes toward PLHIV were associated with HIV testing in bivariable analyses but did not remain statistically significant after adjustment. Marital status and other sexual behavior indicators (recent sexual activity and age at first sexual intercourse) were not included in the final multivariable model due to conceptual overlap with sexual experience (ever had sexual intercourse).

**Table 4:** Factors associated with HIV testing uptake.

Variable	HIV testing uptake			
	Crude OR (95% CI)	*p* value	Adjusted OR (95% CI)	*p* value
Age		<0.001**		<0.001**
15–19	1 (ref:)		1 (ref:)	
20–24	4.51 (3.55, 5.73)		2.57 (1.94, 3.39)	
Gender		0.096		<0.001**
Female	1 (ref:)		1 (ref:)	
Male	1.20 (0.97, 1.50)		1.66 (1.27, 2.18)	
Education		<0.001**		0.004*
No education	1 (ref:)		1 (ref:)	
Primary	1.08 (0.61, 1.93)	0.784	0.49 (0.25, 0.95)	0.034*
Secondary	1.39 (0.80, 2.39)	0.240	0.48 (0.25, 0.92)	0.026*
Higher	3.94 (2.09, 7.40)	<0.001**	0.88 (0.40, 1.96)	0.751
Residence		<0.001**		0.128
Urban	1 (ref:)		1 (ref:)	
Rural	0.52 (0.42, 0.64)		0.81 (0.62, 1.06)	
Marital status		<0.001**		–
Never married	1 (ref:)		–	
Married	3.86 (3.07, 4.85)	<0.001**	–	–
Not currently married or cohabiting^a^	3.24 (1.80, 5.83)	<0.001**	–	–
Occupation		<0.001**		<0.001**
Not working	1 (ref:)		1 (ref:)	
Agriculture/ Self-employed	0.47 (0.32, 0.71)	<0.001**	0.40 (0.26, 0.62)	<0.001**
Clerical/ sales/ services	1.28 (0.91, 1.80)	0.163	0.83 (0.56, 1.22)	0.336
Professional/ technical/ managerial	2.30 (1.56, 3.37)	<0.001**	1.37 (0.92, 2.05)	0.121
Skilled manual	1.11 (0.79, 1.54)	0.546	0.72 (0.48, 1.07)	0.102
Unskilled manual	0.48 (0.34, 0.67)	<0.001**	0.41 (0.29, 0.60)	<0.001**
Wealth quintile		<0.001**		0.045*
Lowest	1 (ref:)		1 (ref:)	
Second	1.53 (1.00, 2.33)	0.049*	1.60 (0.98, 2.61)	0.062
Middle	1.66 (1.13, 2.44)	0.010*	1.62 (1.01, 2.61)	0.046*
Fourth	2.57 (1.75, 3.78)	<0.001**	2.09 (1.27, 3.46)	0.004*
Highest	3.36 (2.28, 4.93)	<0.001**	2.22 (1.31, 3.77)	0.003*
Exposure to mass media		<0.001**		0.072
No exposure	1 (ref:)		1 (ref:)	
Low exposure	2.60 (1.50, 4.51)	0.001*	1.01 (1.10, 3.67)	0.023*
High exposure	2.80 (1.73, 4.51)	<0.001**	1.77 (1.03, 3.03)	0.038
Ever had sexual intercourse		<0.001**		<0.001**
Never	1 (ref:)		1 (ref:)	
Ever	4.24 (3.40, 5.28)		4.59 (3.54, 5.96)	
Recent sexual activity (last 4 weeks)		<0.001**		–
Not currently sexually active	1 (ref:)		–	
Currently sexually active	2.96 (2.39, 3.68)		–	
Age at first sexual intercourse		<0.001**		–
Never had sex	1 (ref:)		–	–
<18 years	3.66 (2.61, 5.13)	<0.001**	–	–
≥18 years	5.02 (3.97, 6.34)	<0.001**	–	–
Comprehensive knowledge of HIV		<0.001**		0.997
No	1 (ref:)		1 (ref:)	
Yes	1.65 (1.29, 2.11)		1.00 (0.72, 1.40)	
HIV-related attitudes toward PLHIV		0.004*		0.093
No discriminatory attitude	1 (ref:)		1 (ref:)	
Discriminatory attitude	0.66 (0.50, 0.88)		0.77 (0.56, 1.05)	

* Significance: *p* < 0.05.

** Highly significance: *p* < 0.001.

a Includes widowed, divorced, and separated/no longer living together.

Odds ratios (ORs) and adjusted odds ratios (AORs) were estimated using survey-weighted logistic regression. Variables shown with estimates only in the crude analysis were examined in bivariable models but were not retained in the final multivariable model due to conceptual overlap and multicollinearity considerations. Model-specific sample sizes vary due to item nonresponse. All analyses accounted for the complex survey design using sampling weights.

## 4 Discussion

In this population-based analysis, we assessed HIV testing uptake and associated factors among 5,185 youth aged 15–24 years in Myanmar using data from the 2015–16 Myanmar Demographic and Health Survey (MDHS).

We observed low levels of HIV testing among youth (10.9%), which were lower than those reported in Burundi (16) and among adolescent girls and young women in Tanzania (17), Zimbabwe (18), and East Africa (11). In Southeast Asia, DHS-based analyses among women indicate substantial variation in HIV testing coverage, with higher uptake among young women in Cambodia and lower uptake in the Philippines (12), compared with the levels observed among youth in Myanmar in the present study. Evidence from global reviews suggests that only around one-third of adolescents and young adults have ever undergone HIV testing, highlighting the limited coverage observed in Myanmar (19). Despite contextual differences, these findings point to persistent regional inequities in HIV testing and the limited reach of services among young people in Myanmar.

Youth aged 20–24 years had higher odds of HIV testing than those aged 15–19 years (AOR = 2.57, 95% CI: 1.94, 3.39, *p* < 0.001), indicating a clear age gradient. This pattern is consistent with DHS-based evidence from The Gambia (20), Papua New Guinea (8), Southeast Asia (12), and Tanzania (17), where HIV testing uptake is higher among older youth. The lower testing uptake among adolescents aged 15–19 years suggests that younger youth remain less reached by existing services, highlighting the need for targeted efforts to improve access to HIV testing for early adolescents in Myanmar. This lower uptake may also reflect developmental and socio-cultural factors in the Myanmar context. Younger adolescents may have limited autonomy in seeking sexual and reproductive health services, and evidence from Myanmar indicates that socio-cultural taboos constrain family communication on these topics, which may limit independent access to HIV testing (21). Taken together, these factors suggest that more systematic integration of HIV testing within youth-oriented sexual and reproductive health services may offer a practical pathway to reach adolescents earlier and more equitably. Male youth had higher odds of HIV testing than females in our study (AOR = 1.66, 95% CI: 1.27, 2.18; *p* < 0.001), contrasting with findings from The Gambia (20), where females had higher testing uptake than males. This pattern suggests a gender-related disparity in HIV testing in Myanmar, highlighting the need to strengthen youth- and female-friendly testing services.

The crude positive association between higher education and HIV testing (OR = 3.94, 95% CI: 2.09, 7.40; *p* < 0.001) attenuated after adjustment, with lower adjusted odds observed for primary (AOR = 0.49, 95% CI: 0.25, 0.95; *p* = 0.034) and secondary education (AOR = 0.48, 95% CI: 0.25, 0.92; *p* = 0.026) and no significant association for higher education. This attenuation indicates that the crude (unadjusted) association was influenced by covariate adjustment and does not reflect an independent association of education with HIV testing uptake. Broad, youth-oriented HIV testing strategies across educational groups may help improve equitable coverage in Myanmar. This finding may be explained by evidence from Myanmar (22) indicating high levels of unmet need for sexual and reproductive health information and HIV testing among youth, suggesting that barriers to access may persist beyond educational attainment, with embarrassment and negative guardian attitudes identified as key barriers. Youth engaged in agriculture/self-employment and unskilled manual work had substantially lower adjusted odds of HIV testing compared with those not working [AORs = 0.40 [95% CI: 0.26, 0.62] and 0.41 [95% CI: 0.29, 0.60]; both *p* < 0.001]. This pattern may reflect occupational disparities in access to or utilization of HIV testing services in the Myanmar context and highlights the need to strengthen youth-oriented HIV testing outreach for working youth.

Sexual experience emerged as the most influential behavioral factor associated with HIV testing. Youth who had ever had sexual intercourse had substantially higher adjusted odds of HIV testing compared with those who had never had sex (AOR = 4.59, 95% CI: 3.54, 5.96, *p* < 0.001). Related indicators of sexual activity (recent sexual activity and age at first sex) showed similar positive associations in bivariable analyses (all *p* < 0.001) but were not retained in the final model due to conceptual overlap. This pattern is supported by evidence among teenage men who have sex with men, where lifetime vaginal or anal sex was associated with higher odds of HIV testing (23). It is also consistent with nationally representative trend data from South Africa, where the proportion ever tested for HIV was consistently higher among those who had ever had sex than among those who had never had sex across survey waves from 2005 to 2017 (24). The strong association between sexual experience and HIV testing uptake suggests that testing remains concentrated among sexually experienced youth in Myanmar. Expanding youth-oriented HIV testing strategies that reach adolescents before or at sexual debut may help improve early testing coverage.

In our study, comprehensive HIV knowledge was associated with HIV testing in bivariable analysis (OR = 1.65, 95% CI: 1.29, 2.11; *p* < 0.001) but not after adjustment. Similar attenuation after adjustment has been reported among young adolescents in Eswatini (7) and among young women in Burundi (16). In contrast, DHS-based analyses across four sub-Saharan African countries reported independent positive associations between comprehensive HIV knowledge and HIV testing among youth (25). Taken together, these mixed findings suggest contextual variability in the role of HIV knowledge, highlighting the need for youth-oriented HIV testing strategies in Myanmar that extend beyond knowledge-based approaches to include broader service delivery and outreach. HIV-related attitudes toward PLHIV are closely linked to HIV knowledge, as better understanding of HIV transmission may reduce stigmatizing beliefs. This may partly explain the attenuation of both variables after adjustment. Similar attenuation was observed for HIV-related discriminatory attitudes, which were associated with lower odds of HIV testing in bivariable analysis in our study (OR = 0.66, 95% CI: 0.50, 0.88, *p* = 0.004) but not after adjustment, consistent with attenuation observed among young women in Burundi (16). This suggests that attitude-related factors may not be independently associated with testing uptake after accounting for covariates, and programmatic efforts should adopt comprehensive, youth-oriented approaches to improve testing coverage.

### 4.1 Limitations and strengths

The cross-sectional design of the MDHS 2015–16 limits any causal inference. Second, self-reported HIV testing and sexual behavior may be affected by recall and social desirability bias, potentially leading to misclassification and conservative estimates. Third, the outcome measured lifetime HIV testing (“ever tested”) and did not capture recency, frequency, or linkage to care, limiting inferences about current testing coverage. Fourth, the MDHS lacks detailed measures of structural and service-related factors of HIV testing among youth, which may result in residual confounding and limit explanation of observed disparities. Fifth, composite measures of HIV knowledge and HIV-related attitudes based on DHS items may not fully capture these constructs, and the strict classification of comprehensive knowledge may mask variation in partial knowledge, potentially attenuating observed associations. Sixth, small unweighted cell counts in some subgroups and the lower proportion of males due to the DHS subsampling design may have reduced the precision of subgroup and sex-specific estimates; thus, these findings should be interpreted with caution. Finally, although the MDHS 2015–16 is the most recent nationally representative survey available for Myanmar, the findings may not fully reflect current HIV testing patterns among youth, highlighting the need for updated youth-focused surveillance. Future research using longitudinal designs and updated nationally representative data with more detailed measures of service access and youth-friendly testing environments would strengthen causal inference and programmatic relevance. This need may be particularly important in rural and underserved ethnic minority settings, where access to HIV testing may be shaped by additional structural, cultural, and linguistic barriers. Complementary qualitative research could further clarify youth-perceived barriers and facilitators of HIV testing in the Myanmar context.

Our study has several strengths. It uses the most recent nationally representative DHS data available for Myanmar and applies appropriate survey-weighted analyses to account for the complex sampling design, enhancing the generalizability of the findings. The focus on youth aged 15–24 years, including both males and females, addresses an important evidence gap in HIV testing research in Myanmar. In addition, the inclusion of youth-relevant behavioral factors, particularly sexual history, alongside sociodemographic variables, provides a more comprehensive assessment of factors associated with HIV testing uptake among youth. The use of standardized DHS measures and transparent analytic procedures enhances the reproducibility of the findings, and the large analytic sample size supports stable multivariable estimates.

### 4.2 Public health implications and recommendations

Given the low uptake of HIV testing among youth in Myanmar and the observed disparities by age, sex, sexual experience, and household wealth, targeted efforts are needed to strengthen youth-friendly HIV testing services, particularly for adolescents aged 15–19 years, working youth, and those from lower socioeconomic backgrounds. In the Myanmar context, this could include expanding community-based and mobile HIV testing in peri-urban and rural areas and strengthening referral linkages between schools, community youth groups, and nearby public or non-governmental testing services to reach younger adolescents. In addition, interventions that address socio-cultural barriers, including stigma, embarrassment, and limited parent-adolescent communication on sexual and reproductive health, may further enhance uptake of HIV testing among youth. The strong concentration of testing among sexually experienced youth highlights the importance of promoting earlier and proactive HIV testing before or at sexual debut, including through school- and community-based platforms and linkages with sexual and reproductive health services. Developing and more systematically integrating HIV testing within broader youth-oriented health services and outreach programs may help reduce structural barriers and improve equitable access to HIV testing among young people in Myanmar.

## 5 Conclusion

Our study indicates that HIV testing uptake among youth aged 15–24 years in Myanmar remains low and varies across key sociodemographic and behavioral characteristics. The marked age gradient and concentration of testing among sexually experienced youth indicate that many adolescents remain unreached by existing testing services. By identifying age-, sex-, socioeconomic-, and behavior-related disparities in HIV testing, this study provides nationally representative evidence to inform more equitable and youth-centered HIV testing strategies in Myanmar. Strengthening early engagement in HIV testing among adolescents is critical to improving timely diagnosis and supporting broader HIV prevention and care efforts.

## Data Availability

The datasets analyzed in this study are not readily available from the authors because the 2015-2016 Myanmar Demographic and Health Survey (MDHS) data are available from the Demographic and Health Surveys (DHS) Program upon registration and approval. Requests to access the datasets should be directed to the DHS Program Data Access portal: https://www.dhsprogram.com/data/available-datasets.cfm.
