# Supplementary material for: Factors associated with HIV testing among youth aged 15–24 years in Myanmar: evidence from the 2015–16 demographic and health survey
Source: Front Reprod Health. 2026 May 4;8:1804929. doi: 10.3389/frph.2026.1804929 (PMC13180833; doi:10.3389/frph.2026.1804929)
Supplement: Supplementary file 1 [file Table1.docx]

**Supplementary table** Bivariate (Chi-square) associations between independent variables and HIV testing uptake (analytic sample, N = 5,185)

| **Variables** | **HIV testing uptake** | | ***p* value** |
| --- | --- | --- | --- |
|  | **No** | **Yes** |  |
|  | **N (%)** | **N (%)** |  |
| **Age** |  |  | < 0.001** |
| 15-19 | 2465 (95.6) | 137 (4.4) |  |
| 20-24 | 2081 (82.7) | 502 (17.3) |  |
| **Gender** |  |  | 0.096 |
| Female | 3266 (89.6) | 461 (10.4) |  |
| Male | 1280 (87.8) | 178 (12.2) |  |
| **Education (n=5184)** |  |  | < 0.001** |
| No education | 314 (92.2) | 24 (7.8) |  |
| Primary | 1175 (91.6) | 133 (8.4) |  |
| Secondary | 2738 (89.5) | 380 (10.5) |  |
| Higher | 318 (74.9) | 102 (25.1) |  |
| **Residence** |  |  | < 0.001** |
| Urban | 1275 (84.3) | 263 (15.7) |  |
| Rural | 3271 (91.2) | 376 (8.8) |  |
| **Marital status** |  |  | < 0.001** |
| Never married | 3475 (93.2) | 269 (6.8) |  |
| Married | 989 (78.2) | 350 (21.8) |  |
| Not currently married or cohabiting^a^ | 82 (80.9) | 20 (19.1) |  |
| **Occupation (n=5172)** |  |  | < 0.001** |
| Not working | 1353 (87.9) | 216 (12.1) |  |
| Agriculture/ Self-employed | 718 (93.9) | 64 (6.1) |  |
| Clerical/ sales/ services | 593 (85.1) | 113 (14.9) |  |
| Professional/ technical/   managerial | 238 (76.1) | 61 (23.9) |  |
| Skilled manual | 521 (86.8) | 86 (13.2) |  |
| Unskilled manual | 1115 (93.9) | 94 (6.1) |  |
| **Wealth quintile** |  |  | < 0.001** |
| Lowest | 866 (94.3) | 62 (5.7) |  |
| Second | 863 (91.6) | 98 (8.4) |  |
| Middle | 1030 (90.9) | 128 (9.1) |  |
| Fourth | 952 (66.6) | 174 (13.4) |  |
| Highest | 835 (83.2) | 177 (16.8) |  |
| **Exposure to mass media** |  |  | < 0.001** |
| No exposure | 488 (95.5) | 35 (4.5) |  |
| Low exposure | 808 (88.9) | 107 (11.1) |  |
| High exposure | 3250 (88.2) | 497 (11.8) |  |
| **Ever had sexual intercourse (n=5143)** |  |  | < 0.001** |
| Never | 3392 (93.8) | 248 (6.2) |  |
| Ever | 1129 (78.0) | 374 (22.0) |  |
| **Recent sexual activity (last 4 weeks) (n=5143)** |  |  | < 0.001** |
| Not currently sexually active | 3701 (91.9) | 373 (8.1) |  |
| Currently sexually active | 820 (79.3) | 249 (20.7) |  |
| **Age at first sexual intercourse (n=5087)** |  |  | < 0.001** |
| Never had sex | 3392 (93.8) | 248 (6.2) |  |
| < 18 years | 392 (80.4) | 115 (19.6) |  |
| ≥ 18 years | 672 (74.9) | 268 (25.1) |  |
| **Comprehensive knowledge of HIV (n=4657)** |  |  | < 0.001** |
| No | 3330 (89.0) | 470 (11.0) |  |
| Yes | 688 (83.0) | 169 (17.0) |  |
| **HIV-related attitudes toward PLHIV (n=4657)** |  |  | 0.004* |
| No discriminatory attitude | 632 (83.9) | 139 (16.1) |  |
| Discriminatory attitude | 3386 (88.7) | 500 (11.3) |  |

**Highly statistical significance: *p* < 0.001. ^a^Includes widowed, divorced, and separated/no longer living together.

Note: Numbers (n) are unweighted counts, and percentages (%) are weighted to represent the national population. P values were obtained from chi-square tests accounting for the complex survey design. Denominators vary by variable due to item nonresponse. Cells with fewer than 25 unweighted cases may yield unstable weighted estimates and should be interpreted with caution.
